# Microplastics, environment and child health

**DOI:** 10.1186/s13052-021-01034-3

**Published:** 2021-03-25

**Authors:** Maria Elisabeth Street, Sergio Bernasconi

**Affiliations:** 1 Division of Paediatric Endocrinology and Diabetology, Paediatrics, Department of Mother and Child, Azienda USL-IRCCS di Reggio Emilia, Viale Risorgimento, 80 42123 Reggio Emilia, Italy; 2 Microbiome Research Hub, University of Parma, Parma, Italy

The substantial increase in scientific studies [1] in recent years has clarified and evidenced that the use of plastic material, widely employed in daily life due to the advantages it offers with respect to other materials, can cause environmental damage. In particular, several studies have focused on microplastics (MPs), defined on the basis of a size smaller than 5 mm. MPs are subdivided into two groups [2]: primary MPs, used both industrially as plastic pellets and in personal care products (i.e. toothpastes, nail polishes, sun creams, scrubs, bath gels) [3] and secondary MPs, derived from the plastic waste dispersed into the environment which undergoes progressive degradation because of photo and thermo-oxidative processes and mechanical abrasion [4]. These latter derive mainly from industrial packaging and textile fibers released into the washing water from machine-washed clothing [2]. Overall, it is estimated that between 75,000 and 300,000 t of microplastics are released into the environment each year in the EU alone [5]. It must also be borne in mind that MPs can release complex mixtures of chemicals into the environment as many types of additives are used in the industrial production of plastics to provide specific features (for example flame retardants, UV stabilizers, heat stabilizers, and plasticizers) [6]. Moreover, due to their hydrophobic surface, MPs can adsorb and concentrate to a high degree hydrophobic organic contaminants (HOCs) such as polycyclic aromatic hydrocarbons (PAHs), organochlorine pesticides and polychlorinated biphenyls (PCBs) [7]. They also accumulate heavy metals such as cadmium, zinc, nickel, and lead [8].

Several studies have shown the presence of MPs in marine waters [9], in terrestrial soil [10], in the air [11] and in tap water [12] in highly populated areas as in regions far from inhabited centers [13]. In other words, MPs are so ubiquitous [14] that scientists have suggested that they will represent an index of the geological era we are experiencing that some geologists define as anthropocene [15]. In addition, various researches have recently shown that MPs can be introduced into the human body, and are found in human organs and tissues [16].

Most commonly MPs are introduced into the body orally, and have been detected in several foods. Most studies have focused on foods of marine origin including invertebrates, crustaceans, and fish [17, 18] but MPs have been found also in sea salt coming from different countries [19], primarily fragments but also filaments and films. Moreover, polyethersulfone and polysulfone have been reported as common types of MPs detected in branded milk samples [20] and have been found in bottled water, honey, beer, plastic teabags and soft drinks in addition [21–23] as well as in fruit and vegetables (particularly in apples and carrots) [24]. Moreover, MPs have been identified in the feces of human volunteers [25]. Based on the consumption of foodstuff, bottled water and on inhalation it has been estimated that a person’s yearly intake in the USA is within a range from 39000 to 121000 particles of MPs [26]. The second mode of introduction of the MPs into the body is through inhalation. Using a Breathing Thermal manikin, Vianello et al. [27] concluded that MPs represent a non-negligible fraction of indoor airborne particulate, which can be both inhaled and ingested. Finally, the possibility of skin absorption should be considered even if there is no definitive evidence to prove this. Further experiences/studies on this aspect would be useful and are warranted [28].

At this stage the logical and fundamental question is: “what are the real risks of disease for humans having ascertained the presence of MPs within the body?” Based on current knowledge this question remains unanswered. However, although currently we are unable to give an exact answer, one must take into consideration the wide range of results obtained from studies in vitro and in animals, including mammals, that have allowed to understand how ingested microplastics pass the intestinal barrier leading to the hypothesis of possible negative effects on human health.

Some recent papers have reviewed in detail the most significant results of this research [29, 30]. As exhaustively summarized by Plata et al. [28], the experimental data have shown that the toxic action occurs by causing: chronic inflammation, changes in the immune response, neurotoxic effects and/or serving as a vector for mycroorganisms and /or toxic chemicals. It should be mentioned also that these actions may require a bioaccumulation phase and do not present in a short frame-time. Moreover, a very unexplored field, that must be investigated, are possible changes induced by MPs on the human microbiota which today is considered of great importance for the effects it can have on various immunological and metabolic disorders [31, 32].

Therefore, today there is a need to develop research on the impact that MPs can have on human health, avoiding easy mass-media alarmism and setting up work based on shared methodologies [33, 34] that also take into account the total exposure (exposome) that an individual can have towards plastic substances in general and /or towards chemical substances contained in plastics but not only in these [35].

As pediatricians, however, we must emphasize that research must also take into account the peculiarities of the developmental age. Children and adolescents have different sensitivity to chemicals than adults and this varies in the different stages of life [36–38]. supporting the need for specific methodologies [39].

Particular attention must be given to fetal life. It has been shown for the first time [40] that MPs can pass the placenta barrier which we already know is permeable to various potentially toxic substances [41].

We know from studies on the Developmental Origins of Health and Disease hypothesis how epigenetic modifications during fetal life can induce disease in adulthood [42–44].

In conclusion, current knowledge on the possible short and long term consequences of exposure to MPs on the health of children and adults should prompt to deepen further the relationships between environment and health. It would be desirable for pediatric scientific societies to take greater responsibility towards environmental issues both at the research level and at the training level of pediatricians.

Finally, it may be useful to remember that even at this time when the viral pandemic is rightly attracting the maximum attention a question to be asked is whether and how the spread of the virus could also be favored by environmental pollution [45, 46]. The necessary use of face-masks has also opened the issue of these being a source of microplastics [47, 48].

## Data Availability

NA.
